# *LZTR1*‐related spinal schwannomatosis and 7q11.23 duplication syndrome: A complex phenotype with dual diagnosis

**DOI:** 10.1002/mgg3.1560

**Published:** 2020-12-02

**Authors:** Karthik Muthusamy, Maciej M. Mrugala, Bernard R. Bendok, Radhika Dhamija

**Affiliations:** ^1^ Department of Clinical Genomics Mayo Clinic Rochester MN USA; ^2^ Department of Neurology Mayo Clinic Phoenix AZ USA; ^3^ Department of Neurologic Surgery Mayo Clinic Phoenix AZ USA; ^4^ Department of Radiation Oncology Mayo Clinic Phoenix AZ USA; ^5^ Division of Hematology/Oncology Department of Internal Medicine Mayo Clinic Phoenix AZ USA; ^6^ Department of Diagnostic Radiology Mayo Clinic Phoenix AZ USA; ^7^ Department of Otolaryngology‐Head and Neck Surgery Mayo Clinic Phoenix AZ USA; ^8^ Department of Clinical Genomics Mayo Clinic Phoenix AZ USA

**Keywords:** 7q11.23 duplication syndrome, dual diagnoses, *LZRT1*, pain, Schwannomatosis

## Abstract

**Background:**

Dual diagnoses in genetics practice are not uncommon and patients with dual diagnosis often present with complex and challenging phenotypes. A combination of meticulous phenotyping and molecular genetic techniques are essential in solving these diagnostic odysseys.

**Methods:**

Clinical features and genetic workup of a patient presenting with incidental schwannomatosis.

**Results:**

A 19‐year‐old male presented with incidental painless schwannomatosis in the background of macrocephaly, distinctive facies, and learning disability. Comprehensive genetic testing with gene panel and chromosomal microarray led to a dual diagnosis of LZTR1‐related schwannomatosis and 7q11.23 duplication syndrome.

**Conclusion:**

We emphasize the need for high index of suspicion and comprehensive genetic testing in complex phenotypes. Interrogation of the interplay between the pathogenic variants in multiple genes could improve our understanding of the pathophysiologic pathways and contribute to therapeutic discoveries.

## Abstract 2

A nineteen year old male presented with incidental painless schwannomatosis in the background of macrocephaly, distinctive facies and learning disability. Comprehensive genetic testing with gene panel and chromosomal microarray led to a dual diagnosis of LZTR1 related schwannomatosis and 7q11.23 duplication syndrome. We emphasize the need for high index of suspicion and comprehensive genetic testing in complex phenotypes. Interrogation of the interplay between the pathogenic variants in multiple genes could improve our understanding of the pathophysiologic pathways and contribute to therapeutic discoveries.

## 1 INTRODUCTION

Dual diagnoses are being increasingly recognized with the advent of next‐generation sequencing and array‐based technologies. Multilocus molecular diagnoses could result in mere addition of the phenotypes when different organ systems are involved, or could lead to an amalgamated phenotype when there are overlapping clinical features (Posey et al., 2017). Atypical clinical manifestations labeled in the past as apparent novel presentation of the disorder might actually represent an additional genetic disorder. Large studies had revealed multilocus molecular diagnoses in a single patient ranging from 2 to 4.9% (Balci et al., 2017; Posey et al., 2017; Smith et al., 2019). In addition to monogenic disorders, copy number variants (CNVs) and single‐nucleotide variants (SNVs) as a part of multiple diagnoses was reported in 11.9% of patients by Posey et al. (2017). Dual diagnosis has also been described to the extent of 0.9% with 22q11.2 syndrome (Cohen et al., 2018).

Schwannomatosis is an autosomal dominant disorder characterized by multiple schwannomas along the peripheral and spinal nerves, and occasional occurrence of unilateral vestibular schwannoma and meningiomas. Around 40% of patients carry an inherited or de novo heterozygous germline pathogenic variation in *LZTR1* (Leucine zipper‐like transcriptional regulator 1) or *SMARCB1* gene (SWItch/Sucrose Non‐Fermentable‐related matrix‐associated actin‐dependent regulator of chromatin subfamily B member 1) (Dhamija et al., 2018). Pain is the most prominent symptom and usually presents in second to fourth decade (Dhamija et al., 2018; Merker et al., 2012).

7q11.23 duplication syndrome is characterized by distinctive facial features with intellectual disability, developmental delay, neurological and behavioral abnormalities, musculoskeletal problems, ascending aortic dilatation, and a peculiar higher threshold for pain perception. Diagnosis is established by detection of recurrent 1.5 to 1.8 MB heterozygous duplication of Williams–Beuren syndrome critical region (WBSCR) (Mervis et al., 2015; Morris et al., 2015).

In this article, we report a patient with dual diagnosis, *LZTR1*‐related Schwannomatosis and 7q11.23 duplication syndrome who presented with incidentally discovered painless schwannomatosis along the spinal nerves. To our knowledge, co‐occurrence of schwannomatosis with other genetic syndromes has not been reported in the past. We postulate that the contrasting pain mechanisms in both the disorders intermixed leading to painless spinal schwannomatosis, which otherwise result in a chronic debilitating pain syndrome.

## 2 CLINICAL REPORT

A 19‐year‐old male presented to Clinical Genomics clinic for evaluation of learning disability, recurrent stereotypic spells from early childhood, and a recent diagnosis of multiple presumed spinal nerve sheath tumors incidentally detected during evaluation of spells. He was born at 38 weeks of gestation with appropriate birth weight. He had hypotonia, global developmental delay, and learning disability noticed since early childhood requiring additional supportive services till about 13 years of age and graduated from high school. There were no birthmarks, seizures, vision, or hearing impairment. Recurrent episodes of stereotyped spells characterized by abrupt loss of consciousness, loss of tone, and fall to the ground were noticed from 3 years of age. Neurology and cardiology evaluation were sought for the spells.

**FIGURE 1:**
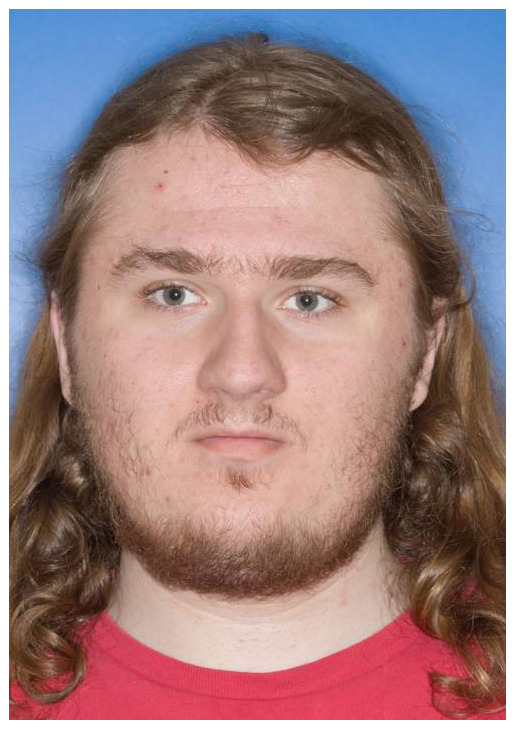
Clinical photograph of the patient: Note the facial features with macrocephaly (OFC: 61 cms), broad forehead, synophrys, straight eyebrows, short philtrum, thin vermilion border of upper lip, and broad chin

On clinical examination, he was right handed and macrocephalic with occipitofrontal circumference of 61 cms (>99 percentile). Distinctive facies (Figure 1) with macrocephaly, broad forehead, synophrys, straight eyebrows, deep‐set eyes, short philtrum, thin vermilion border of upper lip, and broad chin were noted. No definite neurocutaneous stigmata or palpable lumps were noted. General physical examination and neurological examination were normal. Neuropsychological assessment revealed variability in verbal fluency and executive function, with cognitive flexibility scores ranging from average to moderate to severely impaired.

**FIGURE 2:**
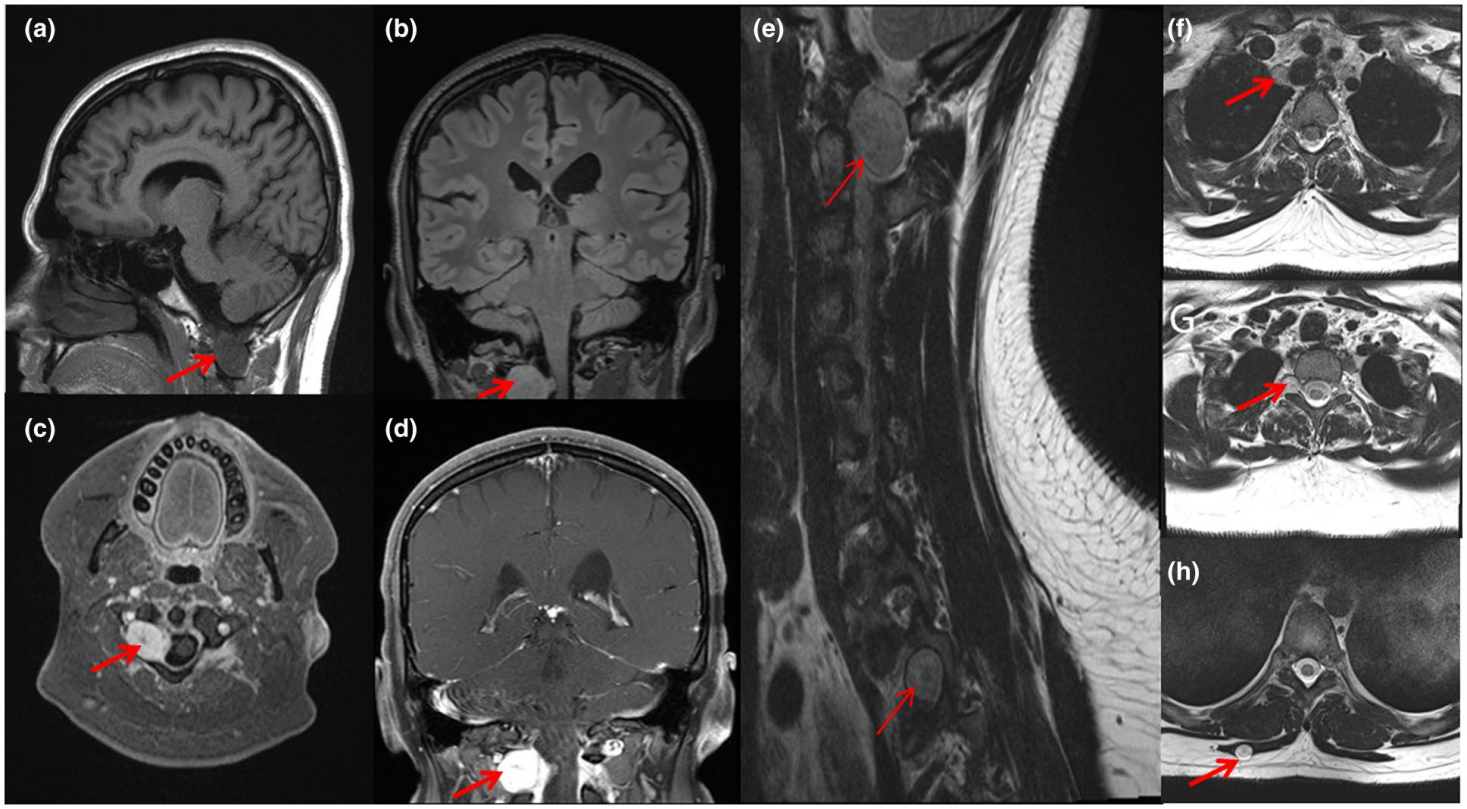
MRI Brain images (a–d) showing T1 hypointense (a), FLAIR isointense (b), and homogenously enhancing lesion (c & d) (arrows) in right C1–C2 region. MRI T2 sagittal section of cervicodorsal spine (e) showing two lesions, one at C1–C2 and the second at T1–T2 (arrows). Also note lesions in T2 axial images visualizing a lesion (arrows) in right superior mediastinum (f), right T1–T2 neural foramen (g), and right paraspinous muscle (h)

MRI brain revealed no intracranial mass or abnormal enhancement. However, an incidental mass was visualized in high cervical region which was further delineated by dedicated spine images. MRI of cervical spine (Figure 2) revealed right C1–C2 level enhancing lesion. Thoracic spine screening revealed a homogeneously enhancing mass within the right T2–3 neural foramen. Additional bilateral extraspinal masses in the paraspinous muscles, and right superior mediastinum were also seen (Figure 2). There was no evidence of an intrinsic spinal cord lesion or abnormal cord signal. Differential diagnosis of schwannomatosis and spinal form of neurofibromatosis type 1 were considered. Initial diagnosis of neurofibromatosis (NF) type 1 was encouraged considering baseline learning disability and macrocephaly, though absence of cafe au lait macules was atypical. Absence of pain and no palpable peripheral schwannomas were considered atypical for schwannomatosis. An independent chromosomal disorder was also considered in view of distinctive facies and learning disability. Hence, schwannomatosis panel and chromosomal microarray were ordered simultaneously.

## 3 METHODOLOGY AND GENETIC FINDINGS

### 3.1 Ethical compliance

The study and testing were approved by institutional ethics committee.

Blood samples were collected clinically from the proband and his parents, and DNA was isolated from blood samples using an Autopure LS automated DNA purifier (Qiagen) following the manufacturer's instructions.

#### 3.1.1 Custom panel sequencing and deletion/duplication analysis, and variant calling

Custom panel sequencing and deletion/duplication analysis of the genes LZRT1, NF1, NF2, and SMARCB1 was performed by GeneDx. Methods from the clinical report are as follows: Genomic DNA from the proband was enriched for the complete coding regions and splice site junctions for genes on this panel using a proprietary capture system developed by GeneDx for next‐generation sequencing with CNV calling (NGS‐CNV). The enriched targets were simultaneously sequenced with paired‐end reads on an Illumina platform. Bidirectional sequence reads were assembled and aligned to reference sequences based on NCBI RefSeq transcripts and human genome build GRCh37/UCSC hg19. After gene‐specific filtering, data were analyzed to identify sequence variants and most deletions and duplications involving coding exons. Alternative sequencing or copy number detection methods were used to analyze region with inadequate sequence or a copy number data by NGS. Reported clinically significant variants were confirmed by an appropriate method. Sequence variants are reported according to the Human Genome Variation Society (HGVS) guidelines. Copy number variants are reported based on the probe coordinates, the coordinates of the exons involved, or precise break points when known. Reportable variants include pathogenic variants, likely pathogenic variants and variants or uncertain significance.

#### 3.1.2 Chromosomal microarray

Chromosomal microarray was performed by Mayo Medical Laboratories using both copy number and single‐nucleotide polymorphism (SNP) probes on a whole‐genome array (Affymetrix CytoScan HD platform).

#### 3.1.3 Genetic findings

Custom panel sequencing and deletion/duplication analysis revealed a heterozygous variant in *LZTR1* gene (NM_006767.3:c.231delA). The frame shift variant is predicted to result in protein truncation or nonsense medicated decay of the transcript p.(Asp78MetfsX23). Loss of function variants in *LZTR1* gene have been previously reported as a known mechanism for disease, and therefore, this variant was interpreted as pathogenic. This variant was inherited from his apparent asymptomatic mother.

Chromosomal microarray revealed a recurrent 1.4 megabase duplication at 7q11.23 consistent with a diagnosis of 7q11.23 duplication syndrome. Genes in the 7q11.23 duplication were *NSUN5*, *TRIM50*, *FKBP6*, *FZD9*, *BAZ1B*, *BCL7B*, *TBL2*, *MLXIPL*, *VPS37D*, *DNAJC30*, *BUD23*, *STX1A*, *MIR4284*, *ABHD11*‐*AS1*, *ABHD11*, *CLDN3*, *CLDN4*, *METTL27*, *TMEM270*, *ELN*, *LIMK1*, *EIF4H*, *MIR590*, *LAT2*, *RFC2*, *CLIP2*, *GTF2IRD1*, *MIR10525*, *GTF2I*, and *LOC101926943*. Both parents tested negative for this duplication, confirming de novo change in the proband.

## 4 DISCUSSION

Dual diagnoses in complex phenotypes are being increasingly recognized. Increasing trend in usage of comprehensive genomic diagnostic testing unmasks independently segregating molecular diagnoses in the same patient with atypical clinical presentations. Overlapping disease phenotypes result in blended clinical manifestations and poses challenge in diagnosis. Such unusual phenotypes are due to independent genes encoding different proteins that interact within the same pathophysiologic pathway (Posey et al., 2017). The Human Phenotype Ontology (HPO) provides a framework for understanding such complex clinical presentations caused by disruption of more than one gene (Robinson et al., 2008). Multiple genetic diagnoses in a single patient were found in 4.9% in the study by Posey et al. Consanguinity and multisystem disease in a proband were commonly found to be associated with multiple diagnoses in a Canadian study (Balci et al., 2017). Apart from monogenic disorders, CNVs and SNVs have also been described as a part of dual diagnoses (Cohen et al., 2018; Posey et al., 2017).

Schwannomatosis is an autosomal dominant tumor suppressor syndrome with reduced penetrance, characterized by predisposition for multiple schwannomas along the peripheral and spinal nerves, and less frequently meningiomas and unilateral vestibular schwannoma (Dhamija et al., 2018; Merker et al., 2012). Germline pathogenic variants in *LZTR1* and *SMARCB1* genes are reported in around 40% of cases (Dhamija et al., 2018; Gonzalvo et al., 2011; Piotrowski et al., 2014). Most common presenting symptom is localized or diffuse pain which becomes chronic and is associated with anxiety and depression leading to poor quality of life (Li et al., 2016; Merker et al., 2012; Ostrow et al., 2017). Spinal schwannomas, increased severity of pain and reduced penetrance were seen more commonly in *LZTR1*‐related schwannomatosis than in *SMARCB1*‐related tumors (Jordan et al., 2018). Germline *LZTR1* pathogenic variants were distributed throughout the gene, and included truncating and out‐of‐frame splicing in 53%, missense in 40%, and splice site variants with in‐frame or unknown effect in 7% (Dhamija et al., 2018; Paganini et al., 2015). Our proband carries a novel frame shift variant in *LZTR1* gene and was inherited from his asymptomatic mother.

7q11.23 duplication syndrome is a recognizable, autosomal dominant syndrome, diagnosed by recurrent 1.5 to 1.8 MB heterozygous duplication of WBSCR. The syndrome is characterized by distinctive facies, variable congenital defects, reduced pain sensitivity, neurodevelopmental, cardiovascular abnormalities, musculoskeletal involvement, and behavioral abnormalities (Mervis et al., 2015; Morris et al., 2015). Facial features described are macrocephaly, broad forehead, straight eyebrows, deep‐set eyes, broad nasal tip, low insertion of columella, short philtrum, thin vermilion of upper lip, high‐arched palate, and ear abnormalities (Mervis et al., 2015). Most neonates have hypotonia and feeding difficulties at birth. Developmental milestones are delayed with motor, speech, and social skills being the prominently affected domains (Morris et al., 2015). Phenotype genotype correlation is poor, except for involvement of elastin and *Gtf2i* genes showing increased risk of aortic dilatation and anxiety disorder, respectively (Mervis et al., 2015). Penetrance is complete and variable expressivity is commonly observed (Mervis et al., 2015; Morris et al., 2015; Patil et al., 2015). Our patient had the classic facial features, developmental issues and was confirmed with a de novo recurrent duplication in 7q11.23 in microarray. Management is primarily supportive and cardiac surveillance is recommended in view of risk of progressive aortic dilatation.

Differential perception of pain is the common overlap in these two disorders. Painful tumors in schwannomatosis secrete factors that act on nearby nerves to augment nociception by producing neuronal sensitization or spontaneous neuronal firing, as evidenced by the work done in cell lines of painful and non‐painful tumors (Ostrow et al., 2019). Increased sensitivity to depolarization by KCl, increased response to noxious TRPV1 and TRPA1 agonists, and upregulation of pain‐associated gene expression were found in the DRG cultures (Ostrow et al., 2019). In 7q11.23 duplication syndrome, one‐fourth of parents reported high pain tolerance in their affected children (Morris et al., 2015). Hypersensitivity to pain has been observed in mice with a deletion of *Gtf2i* gene and hyposensitivity was seen with heterozygous and homozygous duplication of *Gtf2i* gene (Dida et al., 2013). We postulate that the contrariety of pain perception in both the disorders has come together incidentally masking the pain in schwannomatosis, which otherwise presents with a debilitating pain syndrome. In fact patient's baseline pain tolerance was high, history of which was obtained in retrospect.

This report illustrates the challenges in diagnostic evaluation of unusual phenotypes, modification of phenotype in dual or multiple diagnoses, and the need for comprehensive usage of exome and array‐based technologies. The identification of a single‐molecular diagnosis may not be the end of diagnostic odyssey (Smith et al., 2019). Though the analogy contradicts the age‐old teaching of unified diagnosis for a patient, it has value in holistic management and counseling of the family. Understanding of the interaction among multilocus genes in final phenotypic expression will assist us in deciphering pathophysiological pathways and possibly aid in exploring therapeutic options.

## CONFLICTS OF INTEREST

The authors of this manuscript have no conflicts of interest pertaining to the content of this manuscript.

## AUTHOR CONTRIBUTIONS

Karthik Muthusamy drafted the manuscript and prepared figures. Maciej M. Mrugala and Bernard R. Bendok were involved in clinical care and critical revision of the manuscript. Radhika Dhamija conceptualized, critically revised the manuscript and is the corresponding author.
